# The Knowledge of Palliative Care among Geriatric Home Staff in Jordan

**DOI:** 10.4314/ahs.v22i3.25

**Published:** 2022-09

**Authors:** Ahmad M Saleh, Saud M Alrawaili, Mohamed A El-Sakhawy, Walid Kamal Abdelbasset

**Affiliations:** 1 Department of Nursing, College of Applied Medical Sciences, Prince Sattam Bin Abdulaziz University, Al-Kharj 11942, Saudi Arabia; 2 Department of Health and Rehabilitation Sciences, College of Applied Medical Sciences, Prince Sattam bin Abdulaziz University, Al-Kharj 11942, Saudi Arabia; 3 Department of Medical Laboratory Sciences, College of Applied Medical Sciences, Prince Sattam bin Abdulaziz University, Al-Kharj 11942, Saudi Arabia; 4 Department of Physical Therapy, Kasr Al-Aini Hospital, Cairo University, Giza, Egypt

**Keywords:** Knowledge, Palliative Care, Palliative Medicine, Nursing Staff, Cross-Sectional Study, Life Support Care

## Abstract

**Background:**

Palliative care in nursing homes (NHS) is a major challenge, because it gives the demands of the knowledge and skills of nursing staff to provide high-quality care.

**Purpose:**

The purpose of this study was to assess the knowledge of palliative care among nursing home staff.

**Methods:**

A descriptive study design was used, 124 nurses, aged over 30 years and most of them were male working in the nursing home in Jordan. Around 109 participants were responded to the questionnaire. All licensed nurses were included in the study, except of that will be excluded. The knowledge of palliative care was measured via the palliative care survey. knowledge scores were ranged 0–1, with higher scores indicate greater knowledge.

**Results:**

Descriptive statistics was used. The Knowledge of palliative care issues is 0.21 in Jordan (95% confidence interval (CI) 0.19–0.24). Knowledge of physical aspects that can contribute to pain is 0.22 (95% CI 0.2–0.25), and knowledge of psychological aspects that can contribute to pain is 0.21 (95% CI 0.2–0.22).

**Conclusion:**

Education for nursing staff needs to be enhanced to address the specific knowledge gaps. Additional studies with large sample size recommended to explore the effect of educational programs in regard of palliative care in nursing homes.

## Introduction

Palliative care is the active holistic care of individuals across all ages with serious health-related suffering due to severe illnesses, and especially of those near the end of life. It aims to improve the quality of life of patients, their families and their caregivers (Global Consensus Based Palliative Care Definition, 2018; Radbruch et al., 2020). The world's population is ageing, the provision of high-quality palliative care for the elderly people who lived and died in a nursing home is increasing number and it considered as a challenge in the world. Therefore, it requires that staff have the knowledge and skills (DeSA, 2013). In Jordan, the elderly (over 60 years old) is about 4.6% of the population while the percentage is slightly higher for the Arab region as whole (5.6%). The percentage is expected to reach 6.8% in 2025 and 15.3% in 2050 (Tabbarah, 2002). This gradual increase in the elderly population in Jordan as in other Arab countries are expected to cause a shift in the image with increased morbidity and chronic degenerative diseases and increased dependency ratio of old age, retirement, and health care spending (Hafez, Bagchi, & Mahaini, 2000).

More and more people will die of old age after a long life with some chronic life limiting conditions (Etkind et al., 2017). Most parents will experience the need complex and will, at some point, require long-term care in a nursing home (Hall, Petkova, Tsouros, Costantini, & Higginson, 2011) (Marengoni et al., 2011). In Europe, between 12% and 38% of the parents died in a nursing home and the number is expected to increase in future (Reitinger et al., 2013). Furthermore, average survival after admission to the nursing home declined (Forder & Fernandez, 2011), indicates that almost all nursing home residents can be considered at the end of life, making the most appropriate palliative care approach to care for this population. Many residents suffer distressing symptoms such as pain, dyspnea, and depression and have unmet needs of physician communication, emotional support and respect care. Therefore, nursing home meets the needs of palliative care (Lukas et al., 2013)(Takai, Yamamoto-Mitani, Okamoto, Koyama, & Honda, 2010)(Torvik, Kaasa, Kirkevold, & Rustøen, 2009). Education gaps in staff training has been identified as an important barrier to high-quality care (Brazil, Kaasalainen, McAiney, Brink, & Kelly, 2012) (Kupeli et al., 2019).

Literature suggested that nurses may have little knowledge of palliative care, pain and other symptoms control (Wilson, Avalos, & Dowling, 2016) (Gorlén, Gorlén, & Neergaard, 2013) (Watson, Hockley, & Dewar, 2006). Although nurses have much experience in caring for terminally ill residents, they often formal training in palliative care (Prem et al., 2012). Therefore, evidence is required regarding the current knowledge of palliative care nurses in Jordan and factors associated with that knowledge in order to develop effective training strategies.

No previous studies have been conducted to ascertain the knowledge of palliative care staff nursing in Jordan. The purpose of the current study therefore, was to document the current knowledge of palliative care staff nursing (nurses and care assistants) in Jordan theorizing that the nursing staff have acceptable knowledge about palliative care.

## Methods

### Study design

In 2016, the authors conducted a survey in a three public nursing homes in Jordan. In the nursing home, nurses and care assistants usually provide the most highly direct care for elderly residents, each participant of nurses asked to fill a questionnaire about the characteristics of the facility. A quesionnaire on the knowledge palliative care was distributed to all nurses and care assistants who were present in in the nursing home at the time of an investigator of the study visited facility.

### Sampling of nursing homes

Nursing homes were selected by convenience sampling, considering the region, the type of facility and the number of beds, using of public access lists of nursing homes in Amman, Jordan.

### Setting and participants

The term of nursing home in the current study refers to a long-term care center for older people which is defined as “Institutional framework where care is delivered to the elderly who live there, 24 hours per seven days for an indefinite period of time” (Froggatt et al., 2017). In fact, these facilities have different names in different countries, for example, nursing homes, homes for elderly, elderly care center, Geriatric home and Geriatric nursing home. Among those names, there are three types of nursing homes that can be classified into three types: type 1 doctors and nurses with care assistants on site, type 2 nurses with care assistants on site and off site physician and type 3 care assistants onsite and off-site nurses with physician (Froggatt et al., 2017). Participants surveyed in the current study are registered nurses and nursing assistants.

### Data collection Procedure

Supervisors of Geriatric homes received invitation letter from the principal investigator requesting their staff nurses to participate in the study; Initial contact was made by phone and email. In each participating Geriatric nursing home, a coordinator person was identified and named. This coordinator will coordinate to distribute a survey with its' instructions to all nurses and care assistants employed on the nursing home to be returned to him/her immediately after completed. After that coordinator will validate responses and endorse survey to the principal investigator within a month. Anonymity and confidentiality were indicated within informed consent of the survey to all voluntary respondents.

### Measurements

The characteristics respondents were age, gender, professional role, formal training in palliative care and experience in years within geriatric home center.

The knowledge of palliative care was measured using the palliative care knowledge construct. It composed of three sub constructs: (1) psychological items (4 items), reflecting the knowledge of how to manage psychological factors such as anxiety, (2) physical items (5 items), reflecting the knowledge of how to manage physical factors such as pain and (3) end of life items (11 items), reflecting the knowledge of how to administer feeding via tube to prevent aspiration. The items are dichotomous. The Cronbach's alphas for the three subconstructs were as follow psychological (0.97), physical (0.90), and end-of-life factors (0.60). Cronbach alpha for Palliative Care Knowledge (α = 0.81). The items of the palliative care knowledge constructs survey are presented in Table 2. The mean scores on the knowledge subconstructs are between 0 and 1 with higher scores indicating good knowledge (Thompson, Bott, Boyle, Gajewsi, & Tilden, 2011).

**Table 2:** Bivariate correlations of demographic characteristics and knowledge of palliative care

	Age		Years of Experience		Formal Training in Palliative Care	
Knowledge of palliative care	r	p-value	r	p-value	r	p-value
	0.5	0.03*	0.82	0.015*	0.44	0.03*

* p-value < 0.05

### Statistical Analyses

All analyzes were performed using statistical analysis software SPSS 24. Items indicate dependent variables which consist of continuous binary or categorical.

Descriptive statistical analysis was used to describe the sample characteristics. Missing, outliers, and skewness data were checked and handled before analyzing the study data. Data screening was completed for each variable to detect missing, outliers and impossible or extreme score values. Results are shown as estimated differences and 95% confidence intervals (CIs). The level of significance was set at 0.05.

## Results

In the 3 participating geriatric home centers in Jordan, total of 124 staff received a survey. Of these, 109 were returned (88% response rate), 90 (83%) of respondents were nurses – registered nurses and 19 (12%) were assistants care - licensed practical nurses.

### Sample Characteristics

Most respondents (71.5%) were aged over 30, Most of the respondents 79 (72%) were male, followed by 31 (28%) who were female. In addition, 89 (82%) had a baccalaureate level of education and 11 (18 %) had a diploma level of education. The majority 74 (68%) said that they received training and diploma in palliative care, whereas 26 (32%) of respondents said not received any training in palliative care (Table 1).

**Table 1:** Characteristics of the participating nurses and care assistants

Variables	Facility A n = 27	Facility B n = 46	Facility C n = 36
**Age**			
17–35 years	25.9%	17.1%	29.5%
36–50 years	38.1%	52.9%	38.0%
>50 years	36.0%	30.0%	32.5%
**Gender**			
Male	70%	75%	72%
Female	30%	25%	28%
**Professional role**			
Registered nurse	80%	86%	80%
Licensed practical nurse	20%	14%	20%
**Formal training in palliative** **care**			
Yes	75%	60%	70%
No	25%	40%	30%
**Number of years working in** **direct resident care**			
Less than 10 years	46%	38%	29%
More than 10 years	54%	62%	71%

### Knowledge about palliative care

Knowledge nurses about palliative care problems such as breathing, pain and nutrition, were measured by a subscale of “the end of life items” which was 0.21 (95% CI 0.19–0.24). Knowledge of the physical aspects of the disease or treatment that can contribute to pain was measured with sub-scale “physical items” was 0.22 (95% CI 0.2–0.25) in Jordan. Knowledge of psychological factors that can contribute to pain measured with the subscale of “psychological items” was 0.21 (95% CI 0.2–0.22).

**Figure 1:**
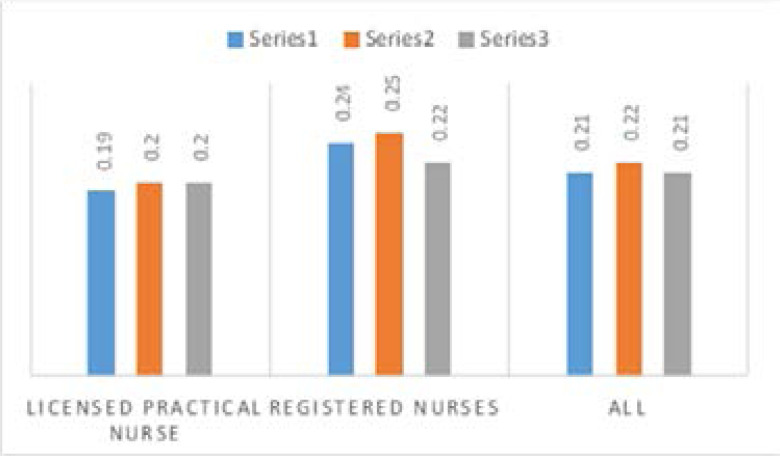
Knowledge of nursing staff about palliative care knowledge construct in all facilities. Data are presented as estimated means and 95% confidence intervals. Scores on the subscale range between 0 and 1 with higher scores indicating greater knowledge.

As presented in Figure 1, Registered Staff nurse's knowledge score did not differ by type of facility (data not shown). However, there are statistically significant differences between registered nurses and licensed practical nurse in all facilities, as registered nurses having a high score of knowledge than the licensed practical nurse in the three subscales (p < 0.05).

### Factors associated with palliative care knowledge

Factors significantly associated with the knowledge of palliative care nurses were age, years of experience and palliative care training reception as part of their basic qualification or a complementary formation (Table 2).

The results showed that there was a significant positive correlation between level of knowledge and age for palliative care, r = 0.5, p < 0.05, (two-tailed). In that, the results indicated, the higher the age of the participants, more knowledge about palliative care will be among them. In addition, better palliative care knowledge was associated years of experience r = 0.82, p < 0.05, (two-tailed). Furthermore, formal training r = 0.44, p < 0.05, (two-tailed).

## Discussion

The results of the current study showed that knowledge about palliative care issues such as pain management or weight loss or the use of feeding tubes, knowledge about psychological and physical factors are generally poor among nurses and nursing assistants in all geriatric nursing home center in Jordan. However, Registered nurses have a better knowledge than the licensed practical nurses, and that applies to those who have formal training conducted in palliative care. This study is considered the first study assessing palliative care knowledge among nurses in geriatric nursing home center in Amman, Jordan. However, the study also has some limitations. The generalizability of the results of the current study could be limited due to some factors, The convenience sample limits the generalizability of the results (Polit & Beck, 2012).

In addition, sample were investigated at only one month and it includes geriatric nursing home center in Amman only not all provinces in Jordan. A survey could be awarded as a further limitation, due to the ability to create biased to social desirability (being to be good), compliance (somewhat) and the trend use extreme notes, the problem with self-reports is that some participants are not an accurate understanding of their own ability or contrast, and reliability of the data obtained by these instruments of self-evaluation dependon the veracity of respondents. In addition to give participants the opportunity to discuss answers of the questions among each other. In consistently, other studies were investigated more than one month to assess palliative care knowledge among staff nurses (Froggatt et al., 2017; (Anstey, Powell, Coles, Hale, & Gould, 2016; Martyn et al., 2019; Manson, Gardiner, & McTague, 2020).

Finally, Palliative care knowledge questionnaire didn't cover aspects like grief, communication and disinheritance within psychosocial construct of the questionnaire, which could be considered important elements for nurses to know about it for geriatric patients. On the other hand, some previous studies cover aspect like grief, communication and disinheritance within psychosocial construct of the questionnaire (Treml et al., 2020; Kimotho, 2018). This study indicates that the science of a palliative nursing care in geriatric nursing home centers shows that there is a decrease level of knowledge among nurses which was congruent with a previous study done by Froggatt et al., 2017. However, there is no evidence of engagement with palliative care initiatives and inside geriatric nursing care homes (Froggatt et al., 2017). This recommend that the level of knowledge about palliative care among staff nurses is affected by many factors, that include accessibility and availability of palliative care services and nursing homes initiatives and scope of national policy.

The result of the current study found that Jordanian staff nurses in geriatric nursing home center have a better understanding than nurses assistants. This result is congruent with a previous literature done in the United States and Australia (Unroe, Cagle, Lane, Callahan, & Miller, 2015). This indicates variations in palliative care education, in addition to a different role and responsibilities (Manson, Gardiner, & McTague, 2020) (Bolt, Meijers, van der Steen, Schols, & Zwakhalen, 2020).

Licensed practical nurses usually work under the supervision of a registered nurses and their responsibilities are limited to provide care. However, most nursing care in nursing homes is provided by the assistants of care, they are in a significant position to record physiological and behavioral changes and to assess and meet the needs of a resident. Therefore, palliative care knowledge is highly relevant for them. So, the current study suggests the need of policies to identify palliative care nursing homes as a framework in which staff, whatever their discipline, the need to know the basic principles of palliative care (Best, Leget, Goodhead, & Paal, 2020).

This implies that all staff levels need to be able to provide high quality care to residents at the end of life (Batiha et al., 2015). In this regard, this study clearly shows that there is great need to invest more in knowledge of palliative care and basic skills, particularly for licensed practical nurse.

The results indicate a low knowledge among nurses in geriatric nursing home and also suggest as a starting point to develop policy regarding the appropriate training. Continuous in-house education and training must also be undertaken with the integration of palliative care curriculum for future nurses (Anstey, Powell, Coles, Hale, & Gould, 2016; Martyn et al., 2019). Management plans are essential to create a work environment that is supportive of staff members developing a major role around palliative care (Anstey et al., 2016). Therefore, Attention to the motivation of the staff, adequate planning and resources should be available to implement new improvement initiatives (Hamdan & Kawafhah, 2015; Holmberg, Hellström, & Österlind, 2019; Esplen et al., 2020).

Other factors affecting the effectiveness of training strategies are in person training, certification of training implementation bedside, easier access to continuing education, coverage for staff to participate in training sessions and opportunities to learn new knowledge and apply new skill methods (Kane et al., 2017; Quan, Lohman, Resciniti, & Friedman, 2019),

The study has some strengths including its consideration the first study assessing palliative care knowledge among nurses in geriatric nursing home center in Amman, Jordan, and high response rate (88%). On other hand, some limitations have been observed in the study such as the convenience sample limits the generalizability of the results, A survey could be awarded as a further limitation, due to the ability to create biased to social desirability, and finally, Palliative care knowledge questionnaire didn't cover aspects like grief, communication and disinheritance within psychosocial construct of the questionnaire, which could be considered important elements for nurses to know about it for geriatric patients.

## Conclusion

This study focused on the knowledge of palliative care among nurses in three facilities of geriatric nursing home centers, being significant professionals in providing care for older people. The findings of the current study indicate low knowledge of basic palliative care issues such as breathing or pain or nutrition. In addition, there are large knowledge variations between them. The provision of adequate training and education in palliative care staff of the nursing home should be high public health priority. It is critical to understand the specific needs of nursing staff in order to develop tailored strategies. Interventions aimed at increasing the competence of nursing staff in providing palliative care for elderly population. Additional studies with large sample size recommended to explore the effect of educational programs in regard of palliative care in nursing homes.
